# Comparative analysis of expression of histone H2a genes in mouse

**DOI:** 10.1186/1471-2164-6-108

**Published:** 2005-08-13

**Authors:** Hiromi Nishida, Takahiro Suzuki, Hiroki Ookawa, Yasuhiro Tomaru, Yoshihide Hayashizaki

**Affiliations:** 1 Laboratory for Genome Exploration Research Group, RIKEN Genomic Sciences Center (GSC), RIKEN Yokohama Institute, 1-7-22 Suehiro-cho, Tsurumi-ku, Yokohama, Kanagawa 230-0045, Japan

## Abstract

**Background:**

At least 18 replication-dependent histone H2a genes are distributed in 3 *Hist *gene clusters on different chromosomes of the mouse genome. In this analysis we designed specific PCR primers for each histone H2a transcript and studied the expression levels and patterns using quantitative RT-PCR (qRT-PCR). In addition, we compared histone H3 K9 acetylation levels in the promoter regions of H2a genes by ChIP (chromatin immunoprecipitation) – quantitative PCR (qPCR) analysis.

**Results:**

RT-PCR analysis indicated that all 20 histone H2a genes assessed in this study are expressed. The replication-dependent histone H2a genes have different expression levels but similar expression patterns. Among the 20 histone H2a genes, the expression-level of *H2afz*, a replication-independent gene, was highest, and that of *Hist1h2aa*, a replication-dependent gene, was lowest. Among 18 replication-dependent H2a genes, the expression level of *Hist3h2a *was highest. The ChIP-qPCR analysis showed that histone H3 K9 acetylation levels in promoter regions of both *H2afz *and *Hist3h2a *are clearly higher than that in the promoter region of *Hist1h2aa*. The H3 K9 acetylation level in the promoter of *Hist1h2aa *is similar to that in the γ-satellite region.

**Conclusion:**

These results strongly suggest that histone H3 K9 acetylation plays a role in the expression of histone genes.

## Background

Eukaryotic genomic DNA is packaged with chromosomal proteins, forming chromatin. The most fundamental repeating unit of chromatin is the nucleosome. The nucleosome core consists of 146 bp of DNA wrapped around an octamer of histone proteins made up of 2 copies each of histones H2A, H2B, H3, and H4, in 1.65 turns [1]. Replication of the eukaryotic chromosomes requires the synthesis of histones to package the newly replicated DNA into chromatin. Control of the level of histone mRNA accounts for much of the control of histone protein synthesis [2]. It is still an open question as to how the expression of individual histone genes is controlled.

The variants and modifications of the histone proteins are related to chromatin structure [3-6]. Specific amino acids within histone tails are targets for a number of post-transcriptional modifications, i.e., acetylation, methylation, phosphorylation, and ubiquitination [3]. In particular, the modification of histone H3 K9 affects chromatin structure. H3 K9 methylation is enriched in transcriptionally silent genes and heterochromatin. On the other hand, H3 K9 acetylation is enriched in transcriptionally active genes [7]. Is this modification related to histone gene expression?

Eighteen replication-dependent histone H2a genes were identified in the mouse genome sequence [8]. Among these 18 genes, 13 are located in the *Hist1 *cluster on chromosome 13, 4 in the *Hist2 *cluster on chromosome 3, and 1 in the *Hist3 *cluster on chromosome 11 [8]. Thus, replication-dependent histone H2a genes are distributed in at least 3 *Hist *clusters. In addition, the mouse has 2 replication-independent histone H2a genes, *H2afx *on chromosome 9 and *H2afz *on chromosome 3. Recently we reported a novel replication-independent histone H2a gene (*H2afj*) on chromosome 6 [9]. *H2afz *and *H2afj *are typical replication-independent genes [9,10]. The H2afz protein is enriched in euchromatic regions and acts synergistically with a boundary element to prevent the spread of heterochromatin [6]. On the other hand, *H2afx *mRNA has both a polyadenylated tail and a stem-loop structure [11], elements typical of, respectively, replication-independent and replication-dependent histone genes.

As cells progress from G1 to S phase, the rate of histone gene transcription increases 3- to 5-fold, and the efficiency of histone pre-mRNA processing increases 8- to 10-fold, resulting in a 35-fold increase in histone protein levels [2,12]. Most promoters of histone genes have CCAAT and TATA boxes [9,13]. Some promoters have an E2F binding motif between the CCAAT and TATA boxes. This E2F binding motif is recognized, and then the E2F transcription factor activates an H2a gene in early S-phase of the cell cycle [14]. However, it is not known how transcription-related proteins cooperate to coordinately regulate histone gene transcription during the cell cycle.

The amino acid sequences of histone H2a proteins are very similar, except for that of H2afz protein [9]. For example, *Hist1h2ab*, *2ac*, *2ad*, *2ae*, *2ag*, *2ai*, *2an*, and *2ao *encode the same structural protein. Among these 8 genes, *Hist1h2ad *and *2ao *have the same nucleotide sequence; however, the others have different nucleotide sequences. Quantitative RT-PCR analysis can be used to show the expression levels of different genes (for example [15]). Thus, in this study we designed the specific PCR primers for each histone H2a gene and studied the expression levels and patterns by qRT-PCR.

## Results and discussion

**Figure 1:**
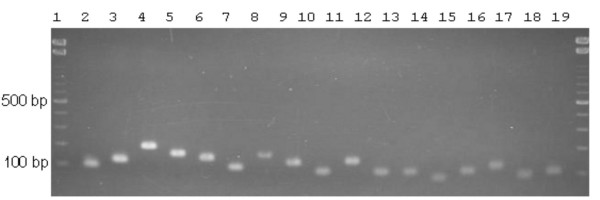
**RT-PCR products. **Lanes 1 and 19, DNA ladder marker; 2, *Hist1h2aa *transcript; 3, *Hist1h2ab *transcript; 4, *Hist1h2ac *transcript; 5, *Hist1h2ad/1h2ao *transcripts; 6, *Hist1h2ae *transcript; 7, *Hist1h2af *transcript; 8, *Hist1h2ag *transcript; 9, *Hist1h2ah *transcript; 10, *Hist1h2ai/1h2aj *transcripts; 11, *Hist1h2ak *transcript; 12, *Hist1h2an *transcript; 13, *Hist2h2aa1/2h2aa2 *transcripts; 14, *Hist2h2ab/2h2ac *transcripts; 15, *Hist3h2a *transcript; 16, *H2afj *transcript [9]; 17, *H2afx *transcript; 18, *H2afz *transcript.

**Figure 2:**
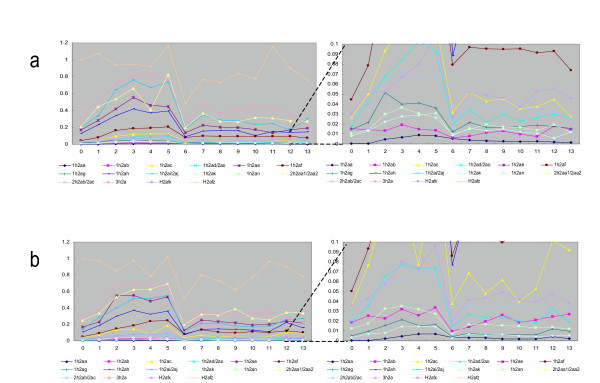
**Expression patterns and levels: results of a) first and b) second qRT-PCR analyses. **X-axis, time (hours); Y-axis, expression level relative to *H2afz *expression level adjusted to 1.0 at 0 h in each experiment.

Each product of the qRT-PCR gave a single band on the agarose gel, located in the expected position (Fig. 1). This result indicates that all histone H2a genes are expressed in Hepa 1–6 cells. The expression levels of 18 replication-dependent histone genes and *H2afx *increased along with cell cycle progression from the beginning (0 h) of S-phase to the middle (2–4 h) of S-phase, and then decreased from the middle to the end (6 h) of S-phase (Fig. 2). On the other hand, the expression level of the replication-independent gene *H2afz *lacked such a single peak during S-phase (Fig. 2).

*H2afz *is regulated in a replication-independent manner, but *H2afx *is regulated in a replication-dependent manner. This pattern is consistent with the results of a previous report that indicated that *H2afx *gives rise to a cell-cycle-regulated mRNA ending in the stem-loop during S-phase, and a polyadenylated mRNA during G1-phase [10]. Therefore, *H2afx *is regulated in a replication-dependent manner (Fig. 2). On the other hand, *H2afz *lacks regulation of a polyadenylated mRNA. Interestingly, expression levels of *H2afz *decreased at the end (6 h) of S-phase, similar to those of replication-dependent genes (Fig. 2). This result suggests that the decrease at the end of S-phase is independent of the histone H2a mRNA structure.

**Figure 3:**
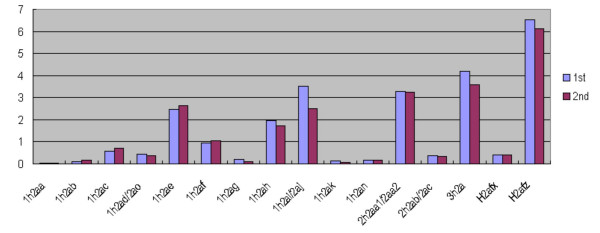
**Sum of expression levels at 0, 1, 2, 3, 4, 5, and 6 h (S-phase) in Fig. 2. **Blue and red indicate a) and b) in Fig. 2, respectively. Y-axis, sum of expression levels.

We compared the sum of expression levels at 0, 1, 2, 3, 4, 5, and 6 h (S-phase) from each histone H2a gene (Fig. 3). Amino acid sequences from the proteins encoded by *Hist1h2ab*, *2ac*, *2ad*, *2ae*, *2ag*, *2ai*, *2an*, and *2ao *were identical. However, among these 8 genes, the expression level of *Hist1h2ae *was 10 to 30 times that of *Hist1h2ag *(Fig. 3). Thus, the expression levels of the genes encoding the same structural protein were different.

Among the 13 genes in the *Hist1 *cluster, the expression level of *Hist1h2ae *was approximately 100 times that of *Hist1h2aa *(Fig. 3). In addition, the 4 genes in the *Hist2 *cluster had different expression levels. Thus, the expression level of *Hist2h2aa1/2aa2 *was approximately 10 times that of *Hist2h2ab/2ac *(Fig. 3). Therefore, the expression levels of genes belonging to the same gene cluster were different.

One possibility is that such different expression levels are caused by different promoters and different binding proteins bound to the promoters. For example, the promoters of *Hist1h2ad*, *Hist1h2af*, *Hist1h2ag*, and *Hist1h2ah *have the E2F binding motif (5'-TTTTCGCGCCC-3') between the CCAAT and TATA boxes [9]. Among these 4 replication-dependent genes, the expression level of *Hist1h2ah *was approximately 10 to 20 times that of *Hist1h2ag *(Fig. 3). In addition, compared among all 20 genes assessed in this paper, the expression levels of *H2afz*, *Hist3h2a*, *Hist2h2aa1/2aa2*, *Hist1h2ae*, and *Hist1h2ai/aj *were higher than that of *Hist1h2ah*, and those of *Hist1h2ak *and *Hist1h2aa *were lower than that of *Hist1h2ag *(Fig. 3). Thus, the relation between the E2F binding motif and the expression level is not clear. Unfortunately, we cannot determine here which structure of the promoters causes such different expression levels.

Next, we compared the histone H3 K9 acetylation levels in the promoter regions of *H2afz *(highest expression), *Hist3h2a *(highest expression among replication-dependent H2a genes), and *Hist1h2aa *(lowest expression). The ChIP-qPCR analysis showed that histone H3 K9 acetylation levels in the promoter regions of both *H2afz *and *Hist3h2a *were clearly higher than that in the promoter region of *Hist1h2aa*. The H3 K9 acetylation level in the promoter of *Hist1h2aa *was similar to that in the γ-satellite heterochromatin region (Table 1). This result indicates that the expression of histone H2a genes is related to the acetylation of histone H3 K9 in the promoter region.

**Table 1:** C_T _values of quantitative PCR for pull-down DNA fragments in ChIP analysis.

	*Hist1h2aa *promoter		*Hist3h2a *promoter		*H2afz *promoter		γ-satellite	
	
	1st	2nd	1st	2nd	1st	2nd	1st	2nd
A: No antibody	26.9	26.95	27.46	27.15	29.45	30.58	8.2	8.17
B: Antibody of H3 K9 acetylated	27.61	27.06	24.14	23.59	26.47	26.68	8.81	8.57
A – B	-0.71	-0.11	3.32	3.56	2.98	3.9	-0.61	-0.4

## Conclusion

This study strongly suggests that histone H3 K9 acetylation plays a role in the expression of histone genes.

## Methods

### Cell cycle synchronization

The cell cycle of mouse Hepa 1–6 cells was synchronized at the end of G1-phase by the addition of thymidine-hydroxyurea. The cell cycle arrest was released by washing out the thymidine-hydroxyurea, then the cells were harvested at intervals of 1 h from 0 to 13 h.

### RNA extraction

Total RNA was extracted by using the RNeasy mini kit (Qiagen) according to the instructions in the manual for the cell line. After that, each sample was treated with DNase I.

### cDNA synthesis

RNA (approximately 0.5 μg) and random hexamer primers were heated to 70°C for 10 min, followed by cooling on ice for 5 min. The cDNA was synthesized in Superscript III First Strand buffer (Invitrogen) according to the manual. The reverse transcriptase was inactivated by a 15-min incubation at 70°C.

### Quantitative PCR

The primers used in this analysis are shown in Table 2. Quantification of GAPDH (glyceraldehyde-3-phosphate dehydrogenase) mRNA (primers 5'-TGTGTCCGTCGTGGATCTGA-3' and 5'-CCTGCTTCACCACCTTCTTGA-3' ; product size 76 bp) was used as a control for data normalization. PCR amplification was performed on an ABI PRISM 7700 Sequence Detection System (Applied Biosystems). The PCR conditions were an initial step of 30 s at 95°C, followed by 40 cycles of 5 s at 95°C and 30 s at 60°C. The SYBR premix Ex *Taq *(Takara) was used according to the manual. Each amplification curve was checked [16]. Expression was assessed by evaluating threshold cycle (C_T_) values. The relative amount of expressed RNA was calculated by using Livak and Schmittgen's method [17]. The qRT-PCR analyses were performed twice. In each analysis, we adjusted the *H2afz *expression level to 1 at 0 h.

**Table 2:** Primers used in this analysis.

Transcript	Sequences (5' to 3'), forward and reverse	Product size (bp)
Hist1h2aa	cggcagtgctagaatacttgaca, gcaggtggcgaggagta	96
Hist1h2ab	gcctgcagttccccgta, atctcggccgtcaggtactc	121
Hist1h2ac	ggctgctccgcaagggt, cttgttgagctcctcgtcgtt	191
Hist1h2ad/2ao	tggacgcggcaagcagggt, agcacggccgccaggtag	162
Hist1h2ae	accggctgctccgcaaa, tgatgcgcgtcttcttgttgt	144
Hist1h2af	cgaggagctcaacaagctgt, ttgggcttatggtggctct	111
Hist1h2ag	tggacgcggcaaacagggc, cagcacggccgccaggtaga	162
Hist1h2ah	atatgtctggacgcggt, acgcgctccgagtagttg	133
Hist1h2ai/2aj	tcgcgccaaggccaagact, cccacgcgctccgagtagtt	102
Hist1h2ak	tacctggcagccgtgcta, cagcttgttgagctcctcgtc	141
Hist1h2an	gaggagctcaacaagctgct, ggtggctctcggtcttcttc	100
Hist2h2aa1/2aa2	aactgtagcccggcccg, ttcgtctgtttgcgcttt	100
Hist2h2ab/2ac	ggcaaagtgacgatcgca, gtggctctcggtcttcttgg	78
Hist3h2a	agcagggcggcaaagctcgt, ttacccttacggagaaggcg	100
H2afx	aaggccaagtcgcgctctt, tcggcgtagtggcctttc	86
H2afz	actccggaaaggccaagaca, gttgtcctagatttcaggtg	100

### Chromatin immunoprecipitation

A total of 2 × 10^7 ^cells were cross-linked with 1% formaldehyde for 10 min at room temperature. First, genomic DNA was cut by micrococcal nuclease. Then it was cut by sonication. The precleared extract was divided into 2 equal portions. One was used for control lacking antibody, and the other was incubated with acetylated histone H3 K9 antibody (Upstate Biotechnology). Following immunoprecipitation, beads were washed in low salt, then high salt, then LiCl, then TE buffers. The qPCR analyses were performed two times. Primers used in quantitative PCR were the *Hist1h2aa *promoter (5'-TTATAGGCGTGGACATT-3' and 5'-CACAGCTTGAATTCCCC-3'), the *Hist3h2a *promoter (5'-CCGCGTTCTTTTCTGAT-3' and 5'-AATTCGTAAGCGCCAGC-3'), and the *H2afz *promoter (5'-GCGCCAATCATCGCTCG-3' and 5'-TCGGGACGCGTCCTTGA-3'). We used γ-satellite as a constitutive heterochromatin. The γ-satellite PCR primers have been reported [18].

## Authors' contributions

HN designed this study and carried out the molecular biological studies. TS and HO carried out the ChIP experiment and qPCR. YT carried out synchronization of cells. YH helped design the study.
